# Longitudinal changes in vitamin D during twin pregnancy: association with maternal–neonatal outcomes

**DOI:** 10.3389/fnut.2025.1667723

**Published:** 2025-11-17

**Authors:** Zhentong Dai, Lin Zhang, Xintong Shi, Yihan Zheng, Shengjia Long, Xinyi Zou, Jun Wei

**Affiliations:** Shengjing Hospital of China Medical University, Shenyang, China

**Keywords:** twin pregnancies, vitamin D, preterm birth, hypertensive disorders of pregnancy, gestational diabetes mellitus, neonate

## Abstract

**Introduction:**

Existing studies suggest vitamin D (VD) deficiency links to adverse pregnancy outcomes in singletons; however, its association with specific adverse outcomes and neonatal health in twin pregnancies remains unclear. This study aimed to explore the relationship between maternal VD levels and maternal and neonatal outcomes in twin pregnancies.

**Methods:**

This study collected VD levels, pregnancy conditions, and neonatal anthropometry from 324 twin pregnancies. Peripheral blood serum was collected from mothers in mid- and late pregnancy to measure VD concentrations. Logistic models assessed VD's association with pregnancy complications and neonatal anthropometry. The restricted cubic splines (RCS) method was used to estimate the risk threshold for spontaneous preterm birth (sPTB). Accounting for sample size and based on the Akaike (AIC) and Bayesian (BIC) information criteria, three knots were placed at the 10th, 50th, and 90th percentiles of the VD distribution. Missing data were handled using sensitivity analyses—including both optimistic and conservative assumptions—and multiple imputation methods.

**Results:**

Among all the participants, the mean mid-pregnancy VD concentration was 25.08 ± 8.31 ng/mL, with 25.6% having sufficient levels, 46.9% insufficient, and 27.5% deficient. Mid-pregnancy VD deficiency independently predicted sPTB (OR: 2.19; 95% CI: 1.07–4.50; *p* = 0.033). The RCS revealed an L-shaped VD-sPTB risk relationship. Each 1 ng/mL VD decrease increased sPTB risk by 14.94% (OR = 0.87). No statistically significant evidence was found between rising VD levels from mid- to late pregnancy and reduced sPTB risk vs. persistent low gestational VD levels. The analysis did not provide evidence for a statistically significant association between maternal mid-pregnancy VD levels and the incidence of hypertensive disorders of pregnancy (HDP), gestational diabetes mellitus (GDM), iatrogenic preterm birth (iPTB), or neonatal birth weight (all *p* > 0.05). In contrast, chorionicity (*p* = 0.004), HDP (*p* = 0.046), and neonatal sex (*p* = 0.021) were significant determinants of birth weight.

**Conclusions:**

In twin pregnancies, maintaining adequate VD levels during mid-pregnancy represents a critical preventive window for reducing sPTB. For women with deficiency at this stage, the timing of supplementation may be more critical than dose. Although maternal VD status shows no significant association with neonatal anthropometry, the continued monitoring and optimization of VD levels throughout pregnancy remain essential for maternal–neonatal health.

## 1 Introduction

Vitamin D (VD), a fat-soluble vitamin, not only regulates calcium–phosphorus metabolism and bone formation but also modulates cell proliferation, differentiation, and apoptosis (1). It plays significant roles in the immune, respiratory, endocrine, and nervous systems (2–5). In singleton pregnancies, VD deficiency correlates with gestational diabetes mellitus (GDM), hypertensive disorders of pregnancy (HDP), and preterm birth (PTB) (6–10). Twin pregnancy studies show inconsistent results: some link VD sufficiency to reduced GDM risk (11), while others find no association (12). VD may influence HDP through placental and RAAS pathways (13, 14), though conflicting evidence exists (15). PTB (< 37 weeks) affects 9.9% of pregnancies globally, with twin pregnancies having 58.7% PTB rates due to uterine overdistension, HDP/GDM risks, and twin-specific complications. VD-PTB correlations remain debated, with studies reporting increased risk (16–18), no association (19–21), or paradoxical outcomes (22). Rising twin pregnancy rates (3.69%) in China from assisted reproduction amplify these challenges. Regarding the relationship between maternal VD levels during pregnancy and neonatal anthropometric parameters, although VD is known to influence skeletal development, two studies focusing on twin pregnancies found no significant association between maternal VD levels and neonatal birth length (1, 23).

This study aimed to assess the VD levels in mid-pregnancy for twin pregnancies; investigate their associations with GDM, HDP, and PTB; evaluate whether targeted VD supplementation in late pregnancy could reduce complication risks; and determine the relationship between maternal VD status and neonatal anthropometric measurements.

## 2 Materials and methods

### 2.1 Study population

This retrospective cohort study enrolled twin pregnancies who underwent antenatal care and were delivered at Shengjing Hospital of China Medical University, between January 2022 and November 2024.

**Figure 1:**
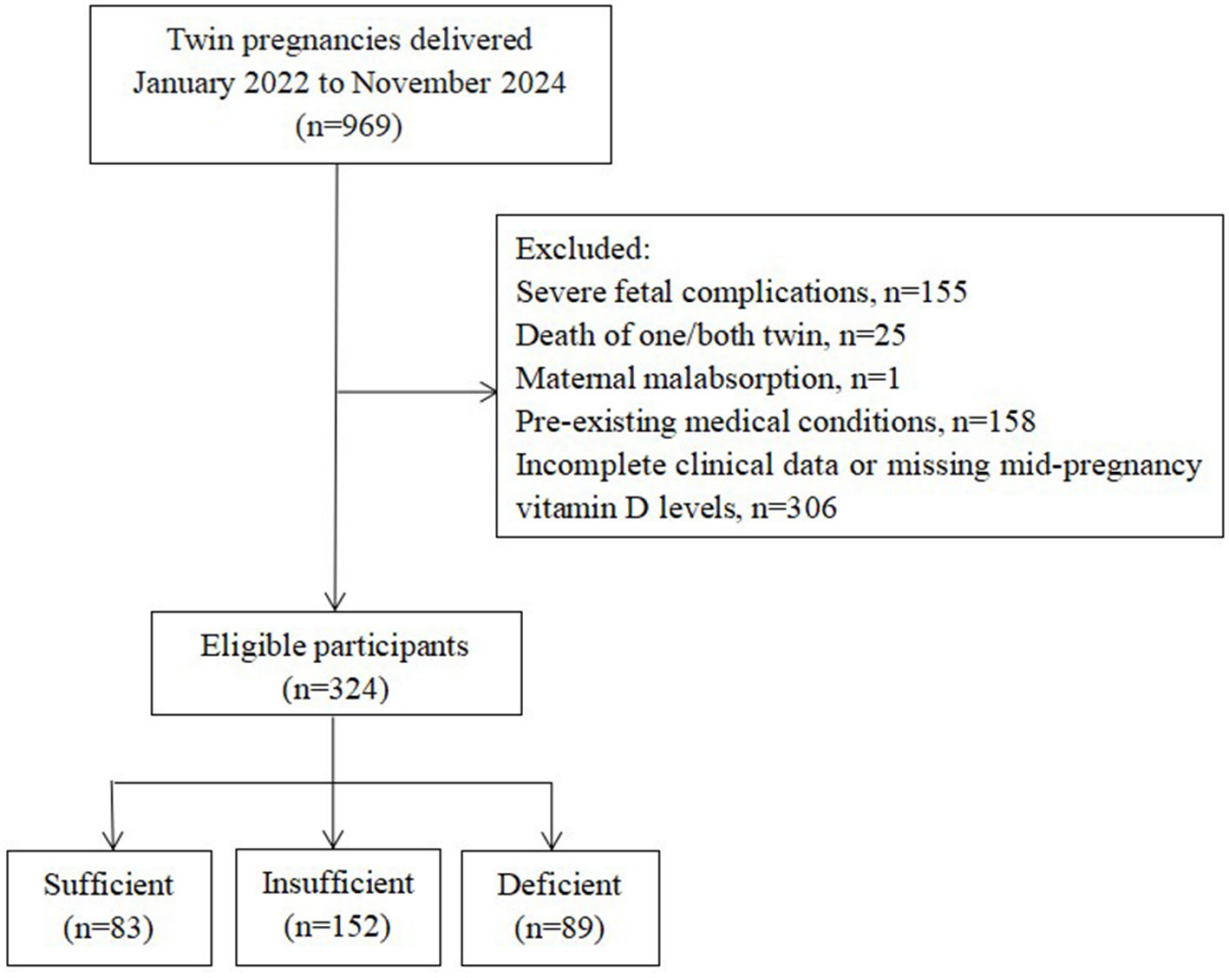
Flowchart showing selection of participants included in this analysis.

Our study included pregnant women with twin pregnancies from whom peripheral blood samples had been collected during the mid-pregnancy to measure VD levels, who were followed during subsequent regular prenatal check-ups, and who had complete delivery records archived at our hospital. The exclusion criteria included the following: pre-pregnancy comorbidities, such as chronic hypertension, type 1 or type 2 diabetes, hyperthyroidism, and autoimmune diseases; maternal malabsorption disorders, such as coeliac disease, inflammatory bowel disease, and previous gastrointestinal surgery (e.g., partial gastrectomy); and severe fetal anomalies or complications, such as twin-to-twin transfusion syndrome and single or double intrauterine fetal demise. The final analysis comprised 324 eligible twin gestations (Figure 1).

The study protocol was approved by the Ethics Committee of Shengjing Hospital of China Medical University (Approval No.: 2021PS444K).

### 2.2 Diagnosis

PTB was defined as delivery occurring prior to 37 completed weeks of gestation or fewer than 259 days following the first day of the last menstrual period. PTB was further classified into spontaneous preterm birth (sPTB) and iatrogenic preterm birth (iPTB). sPTB encompassed both preterm labor with intact membranes (e.g., resulting from premature cervical maturation triggering uterine contractions) and preterm prelabour rupture of membranes (PPROM). In contrast, iPTB referred to the deliberate termination of pregnancy via the induction of labor or cesarian delivery prior to 37 weeks' gestation, which was undertaken when maternal or fetal health complications precluded the continuation of pregnancy. HDP refers to women with gestational hypertension and women presenting with pre-eclampsia. GDM refers to diabetes diagnosed in the mid- or late pregnancy that was not clearly present before gestation, in women with previously normal glucose metabolism. The diagnosis is established using a 75 g oral glucose tolerance test (OGTT) performed between 24 and 28 weeks of gestation. The diagnostic thresholds for the 75 g OGTT are as follows: fasting plasma glucose ≥5.1 mmol/L, 1-h post-load glucose ≥10.0 mmol/L, and/or 2-h post-load glucose ≥8.5 mmol/L. GDM is diagnosed if any single glucose value meets or exceeds these criteria.

### 2.3 Vitamin D measurement and grouping

Maternal peripheral blood specimens were acquired during two gestational periods—mid-pregnancy (16–28 weeks) and late pregnancy (>30 weeks)—employing clot activator tubes for standardized sample processing. The serum was centrifuged at 5,600 rpm for 10 min at room temperature. The total VD levels were quantified via chemiluminescence immunoassays by summing the 25(OH)D_3_ and 25(OH)D_2_ concentrations. The intra-assay coefficient of variation was ≤ 8.9%, and the inter-assay coefficient of variation was ≤ 10.8%, with no significant batch effect observed. The participants were stratified into three VD status groups as per the Endocrine Society's guidelines: deficiency (< 20 ng/mL), insufficiency (20–30 ng/mL), and sufficiency (≥30 ng/mL).

### 2.4 Data collection

The collection of participant-related data primarily encompassed three categories: firstly, demographic characteristics, mainly including age, employment status, place of residence, and nulliparity; secondly, clinical covariates, primarily encompassing VD levels during pregnancy, chorionicity, mode of conception, pre-pregnancy weight, pre-pregnancy body mass index (BMI), and gestational week at delivery; and thirdly, variables of other classifications, including the participant's height, weight at delivery, BMI at delivery, obstetric complications, as well as the neonate's length, head circumference, chest circumference, and weight.

The maternal-related data were gathered primarily through four avenues: information such as employment status and place of residence was obtained via self-reporting by the study participants; data such as VD levels during pregnancy, chorionicity, and mode of conception were sourced from medical record abstraction; measurements such as participant height and weight at delivery were obtained through clinical measurement; and values such as BMI were derived computationally from the collected data. Neonatal measurements were obtained using a standardized method by trained medical staff within 1 h of delivery. Birth weight was measured without a nappy using a calibrated electronic scale.

### 2.5 Missing data handling

Among the 263 sPTB and term delivery cases, late-pregnancy VD data were missing in 21.7% (57/263), which were identified as missing-at-random (MAR) via statistical testing. Multiple imputation was performed to generate 10 datasets to minimize bias, with the pooled results being statistically analyzed, following by sensitivity analysis to enhance the robustness of the conclusions.

### 2.6 Statistical analysis

Normally distributed continuous variables are expressed as means ± standard deviations, with between-group comparisons conducted via ANOVA; if significant differences were detected, *post hoc* pairwise analyses were performed using the Least Significant Difference (LSD) method. Categorical variables are presented as counts (percentages) and were analyzed using chi-square or Fisher's exact tests. Multivariable binary logistic regression and multivariate logistic regression were used to estimate the adjusted odds ratios (ORs) and 95% confidence intervals (CIs) for VD-associated pregnancy complications (statistical significance: *p* < 0.05). The restricted cubic splines (RCS) method was used to assess non-linearity between VD and sPTB, with three knots placed. The threshold VD concentration corresponding to an OR of 1 was identified. The increase in risk was derived from the OR value, whereby the reciprocal of the odds ratio obtained through logistic regression was used to determine the percentage increase in risk. Missing data were addressed via multiple imputation. Multivariable linear regression was used to assess the relationships between maternal VD and neonatal anthropometry.

## 3 Results

### 3.1 Baseline characteristics including outcome measures among participants with different vitamin D levels

The overall mean 25(OH)D concentration was 25.08 ± 8.31 ng/mL. The baseline characteristics including outcome measures of pregnant women with different VD levels are presented in Table 1. From the perspective of baseline characteristics, comprehensive analysis revealed that among 324 participants, 25.62% had sufficient VD levels, 46.91% had insufficient VD levels, and 27.47% were deficient in VD. Our cohort showed that individuals conceiving through assisted reproductive technology (ART) and those with dichorionic twin pregnancies had higher VD levels compared to other subgroups, with significantly elevated levels observed in individuals whose blood samples were collected during summer and autumn. From the perspective of pregnancy complications as an outcome measure, a clinically meaningful variation was observed in the incidence of sPTB (*p* < 0.001). The incidence of sPTB was significantly higher among VD-deficient women (51.69%) compared to those with sufficient (30.12%) and insufficient (27.63%) VD levels.

**Table 1:** Baseline characteristics including outcome measures of participants, stratified by VD levels.

**Variable**	**Sufficient group (*n* = 83)**	**Insufficient group (*n* = 152)**	**Deficient group (*n* = 89)**	***p*-*Value***
**Age**				0.401
< 35 years, *n* (%)	53 (63.9)	107 (70.4)	65 (73.0)	
≥35 years, *n* (%)	30 (36.1)	45 (29.6)	24 (27.0)	
**Employment status**				0.451
Employed, *n* (%)	64 (77.1)	114 (75.0)	73 (82.0)	
Unemployed, *n* (%)	19 (22.9)	38 (25.0)	16 (18.0)	
**Chorionicity**				**0.004**
Monochorionic, *n* (%)	11 (13.3)	31 (20.4)	30 (33.7)	
Dichorionic, *n* (%)	72 (86.7)	121 (79.6)	59 (66.3)	
**Conception method**				**0.030**
Natural Conception, *n* (%)	28 (33.7)	60 (39.5)	47 (52.8)	
Assisted Reproduction, *n* (%)	55 (66.3)	92 (60.5)	42 (47.2)	
**Parity**				0.240
Multiparous (≥1), *n* (%)	6 (7.2)	14 (9.2)	13 (14.6)	
Nulliparous (0), *n* (%)	77 (92.8)	138 (90.8)	76 (85.4)	
**Residence**				0.927
Rural, *n* (%)	8 (9.6)	15 (9.9)	10 (11.2)	
Urban, *n* (%)	75 (90.4)	137 (90.1)	79 (88.8)	
**Pre-pregnancy BMI**				0.390
< 18.5kg/m^2^, *n* (%)	6 (7.2)	7 (4.6)	8 (9.0)	
≥18.5kg/m^2^, *n* (%)	77 (92.8)	145 (95.4)	81 (91.0)	
**Sampling season**				**0.047**
Summer/Autumn, *n* (%)	33 (39.8)	78 (51.3)	52 (58.4)	
Winter/Spring, *n* (%)	50 (60.2)	74 (48.7)	37 (41.6)	
HDP, *n* (%)	27 (32.53)	32 (21.05)	18 (20.22)	0.093
GDM, *n* (%)	25 (30.12)	47 (30.92)	23 (25.84)	0.693
PTB, *n* (%)	46 (55.42)	68 (44.74)	60 (67.42)	**0.003**
sPTB, *n* (%)	25 (30.12)	42 (27.63)	46 (51.69)	**< 0.001**
iPTB, *n* (%)	21 (25.30)	26 (17.11)	14 (15.73)	0.209

Bold *P*-values indicate that the value is less than 0.05.

### 3.2 The correlation between maternal mid-pregnancy vitamin D levels and common pregnancy complications

We performed multivariate logistic regression and multivariable binary logistic regression to determine the correlation between maternal mid-pregnancy VD levels and pregnancy complications, as detailed in Table 2. According to either the unadjusted or the adjusted model, no statistically significant association was observed between maternal mid-pregnancy VD levels and the risk of GDM, HDP, or iPTB. Compared to participants with sufficient VD levels, those with VD deficiency exhibited a significantly higher risk of sPTB (OR: 2.35; 95% CI: 1.18–4.67; *p* = 0.015). After adjusting for confounders including maternal age, chorionicity, conception method, employment status, primiparity, pre-pregnancy BMI, and residential area, the association between VD deficiency and sPTB remained significant (adjusted OR: 2.19; 95% CI: 1.07–4.50; *p* = 0.033). Supplementary Table S1 presents the full results of the associations between all confounding factors and VD levels with sPTB outcomes. It is noteworthy that no statistically significant differences were observed in any of the collected baseline characteristics among the three groups (sPTB, iPTB, and term birth) (all *p* > 0.05), indicating good comparability between the groups, while significant differences in VD levels were evident, as shown in Supplementary Table S2.

**Table 2:** Association between vitamin D status and maternal pregnancy complications and obstetric outcomes.

**Vitamin D levels**	**Crude OR (95% CI)**	***p*-*Value***	**Adjusted OR^a^ (95% CI)**	***p*-*Value***
**HDP**				
Sufficient (≥30 ng/mL)	1.000		1.000	
Insufficient (20–30 ng/mL)	0.553 (0.303–1.010)	0.054	0.562 (0.301–1.051)	0.071
Deficient (< 20 ng/mL)	0.526 (0.263–1.050)	0.069	0.637 (0.303–1.338)	0.234
**GDM**				
Sufficient (≥30 ng/mL)	1.000		1.000	
Insufficient (20–30 ng/mL)	1.038 (0.581–1.858)	0.899	1.075 (0.590–1.958)	0.813
Deficient (< 20 ng/mL)	0.808 (0.415–1.576)	0.532	0.904 (0.444–1.841)	0.781
**PTB**				
Sufficient (≥30 ng/mL)	1.000		1.000	
Insufficient (20–30 ng/mL)	0.687 (0.401–1.176)	0.171	0.669 (0.388–1.155)	0.149
Deficient (< 20 ng/mL)	1.664 (0.896–3.092)	0.107	1.632 (0.852–3.127)	0.140
**sPTB**				
Sufficient (≥30 ng/mL)	1.000		1.000	
Insufficient (20–30 ng/mL)	0.740 (0.395–1.387)	0.348	0.708 (0.374–1.338)	0.287
Deficient (< 20 ng/mL)	2.348 (1.180–4.671)	**0.015**	2.190 (1.066–4.499)	**0.033**
**iPTB**				
Sufficient (≥30 ng/mL)	1.000		1.000	
Insufficient (20–30 ng/mL)	0.545 (0.273–1.091)	0.086	0.545 (0.270–1.102)	0.091
Deficient (< 20 ng/mL)	0.851 (0.370–1.956)	0.703	0.902 (0.378–2.155)	0.817

^a^Adjusted for maternal age, chorionicity, conception method, employment status, parity, pre-pregnancy BMI and residence. Bold *P*-values indicate that the value is less than 0.05.

**Figure 2:**
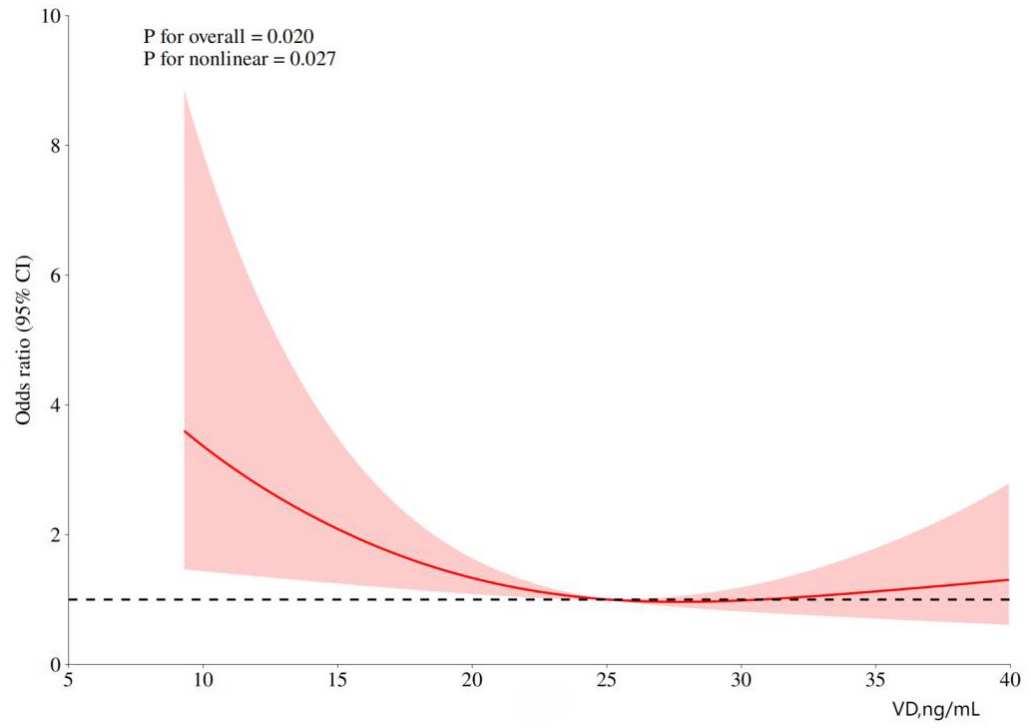
RCS curve of the relationship between vitamin D levels and spontaneous preterm birth.

To further determine the magnitude of this association, RCS modeling was performed to compare sPTB and term cohorts (*n* = 263). The RCS curve revealed an L-shaped association between the mid-pregnancy VD concentrations and risk of sPTB. After adjusting for confounders, this non-linear relationship persisted (*p* for overall association = 0.020; *p* for non-linearity = 0.027), as shown in Figure 2. The inflection point of the curve intersecting with OR = 1 occurred at 25.06 ng/mL, indicating that mid-pregnancy VD levels < 25.06 ng/mL in twin pregnancies were a risk factor for sPTB. For VD levels ≥ 25.06 ng/mL, the 95% CI of the curve included OR = 1, suggesting no additional protective effect at higher concentrations.

Using 25.06 ng/mL as the threshold, the 263 twin pregnancies were reclassified into subgroups. Among women with VD levels < 25.06 ng/mL (*n* = 131), 61 experienced sPTB, yielding an absolute risk of 46.56%. In the group with VD levels ≥25.06 ng/mL (*n* = 132), 52 cases of sPTB were recorded, corresponding to an absolute risk of 39.40%. When the mid-pregnancy VD levels were analyzed as a continuous variable in binary logistic regression that had been adjusted for age, mode of conception, chorionicity, primiparity, and pre-pregnancy BMI, the results demonstrated a significant association (*p* = 0.001; OR = 0.87), as shown in Supplementary Table S3. This indicates that every 1 ng/mL decrease in VD levels below 25.06 ng/mL was associated with a 14.94% increase in the relative risk of sPTB.

### 3.3 Relationship between vitamin D improvement and spontaneous preterm birth risk

Among the 263 twin pregnancies (sPTB and term birth groups), 57 lacked late-pregnancy VD retesting. Of the remaining 206 participants, 103 were subsequently allocated to two distinct groups based on their mid- and late-pregnancy VD levels relative to the 25.06 ng/mL threshold:

1. Group 1: Both mid- and late-pregnancy levels < 25.06 ng/mL (*n* = 61, 59.22%).
2. Group 2: Mid-pregnancy level < 25.06 ng/mL; late-pregnancy level ≥ 25.06 ng/mL (*n* = 42, 40.78%).

To evaluate whether targeted VD improvement reduces the risk of sPTB, the sPTB rates were compared between Groups 1 and 2. As shown in Table 3, no significant difference was observed (40.5% vs. 44.3%; *p* = 0.703). Meanwhile, we conducted a binary logistic regression analysis to examine the association between changes in VD levels and sPTB. With Group 1 as the reference and after adjusting for confounding factors, including age, conception method, chorionicity, parity, residence, pre-pregnancy BMI, employment status, and mid-pregnancy VD levels, elevated VD levels demonstrated no statistically significant protective effect against sPTB (*p* = 0.462).

**Table 3:** A comparison and correlation analysis of spontaneous preterm birth rates between Groups 1 and 2.

**Group**	**Total *n* (%)**	**sPTB Rate (%)**	***p*-*Value***	**Crude OR** ** (95% CI)**	***p*-*Value***	**Adjusted OR^a^** ** (95% CI)**	***p*-*Value***
Group 1	61 (59.22)	44.26	0.703	1.000		1.000	
Group 2	42 (40.78)	40.48		0.856 (0.386–1.900)	0.703	1.429 (0.552–3.699)	0.462

^a^Adjusted for maternal age, chorionicity, conception method, employment status, parity, pre-pregnancy BMI and residence.

The above findings revealed that among pregnant women with mid-pregnancy VD levels < 25.06 ng/mL, a subsequent elevation of VD levels to ≥25.06 ng/mL during late pregnancy did not significantly reduce the risk of sPTB. A sensitivity analysis was conducted to account for missing data from 57 participants. Under the conservative assumption (all 57 participants had late-pregnancy VD levels < 25.06 ng/mL) and the optimistic assumption (all 57 participants had late-pregnancy VD levels ≥ 25.06 ng/mL), results presented in Supplementary Table S4 showed no statistically significant differences in sPTB incidence between the two groups under either scenario. After adjusting for confounding factors, multivariable binary logistic regression indicated that elevated VD levels were not significantly associated with the risk of sPTB in either case. Additionally, we performed multiple imputation to generate 10 imputed datasets, which yielded consistent results.

Both the sensitivity analyses and multiple imputations demonstrated that elevated VD levels in late pregnancy do not reduce the risk of sPTB.

### 3.4 Correlation of maternal vitamin D levels with neonatal outcomes

Table 4 reflects the characteristics of the newborns. Table 5 presents the linear regression analysis of neonatal birth weight after adjustment for relevant confounding factors. A higher gestational age at delivery, greater maternal height, higher pre-pregnancy BMI, and faster gestational weight gain rate were positively correlated with neonatal birth weight.

**Table 4:** Characteristics of the newborns.

**Characteristic**	**Overall**
**Sex of the newborn**	
Male, *n* (%)	330 (50.93)
Female, *n* (%)	318 (49.07)
Neonatal weight, g	2,407.94 ± 424.58
Birth weight discordance, *n* (%)	38 (11.73)

**Table 5:** Association between pregnant covariates and neonatal birthweight.

**Variable**	**B**	**Beta**	**95% CI**	***p*-*Value***
Maternal mid-pregnancy VD	−0.608	−0.012	−3.778 to 2.563	0.706
Maternal age	1.831	0.016	−5.103 to 8.764	0.604
Maternal height	9.719	0.112	4.439 to 14.999	**< 0.001**
Maternal pre-pregnancy BMI	14.567	0.121	7.117 to 22.016	**< 0.001**
Maternal gestational weight gain rate	321.959	0.140	179.298 to 464.621	**< 0.001**
Gestational age at delivery	176.543	0.767	162.305 to 190.782	**< 0.001**
GDM	−10.506	−0.011	−67.156 to 46.143	0.715
HDP	−62.505	−0.063	−124.000 to −1.009	**0.046**
**Chorionicity**				
Monochorionic	Reference			
Dichorionic	101.406	0.099	32.398 to 170.414	**0.004**
**Newborn sex**				
Female-female twin pairs	Reference			
Sex-discordant twin pairs	48.675	0.056	−19.675 to 117.250	0.162
Male-male twin pairs	77.047	0.085	11.665 to 142.430	**0.021**

Analyses were adjusted for all confounding factors listed above. The unstandardized coefficient (B) represents the mean change in birth weight (in grams) associated with each unit change in the independent variable. Bold *P*-values indicate that the value is less than 0.05.

Compared to normotensive pregnant women, those with HDP delivered newborns with lower birth weights, averaging a reduction of approximately 63 grams. Neonates from dichorionic pregnancies exhibited higher birth weights than those from monochorionic pregnancies. Further analysis of the interaction effect of chorionicity revealed no significant difference in the impact of mid-pregnancy maternal VD levels on fetal weight. Male–male twin pairs had a significantly higher mean birth weight compared to female–female twin pairs (mean increase: 77 grams; *p* = 0.021). However, comparative analysis revealed comparable neonatal weights across sex-discordant and same-sex female twin pairs (*p* = 0.162).

We conducted linear regression analyses on maternal mid-pregnancy VD levels and neonatal head circumference, chest circumference, and body length; as shown in Supplementary Table S5, the results demonstrated no significant correlations between VD levels and these anthropometric parameters.

## 4 Discussion

VD deficiency is highly prevalent in the general population and even more common among pregnant women, constituting a significant public health concern. This study found that maintaining adequate VD levels during mid-pregnancy in twin pregnancies reduces sPTB risk, identifying a critical prevention window. There is a saturation effect in the protective role of mid-pregnancy VD levels against sPTB. That is, once maternal VD levels reach a threshold, higher levels do not provide additional protective effect. Linear correlation analysis revealed that neonatal birth weight in twin pregnancies was not associated with maternal mid-pregnancy VD levels. Apart from gestational age at delivery, maternal height, and gestational weight gain rate, birth weight was primarily influenced by chorionicity, HDP, and neonatal sex.

This study reports higher twin pregnancy VD levels than singleton levels (24, 25), aligning with prior research (11, 12). Potential explanations for the higher VD levels in twin pregnancies include the following: The larger placental surface area in twin pregnancies may enhance 1α-hydroxylase production, promoting VD activation. Maternal physiological adjustments to meet dual fetal nutritional demands—potentially through increased VD synthesis or reduced catabolism—may sustain higher circulating levels. Twin-bearing mothers may prioritize nutritional supplementation and outdoor activities, collectively improving their VD status. In addition, we observed that naturally conceived twins exhibited lower VD levels compared with those who were conceived via assisted reproductive technology. This disparity may reflect stricter preconception nutritional guidance, personalized supplementation protocols, frequent monitoring, and antioxidant regimens (to enhance the oocyte/embryo quality) in assisted reproductive technology pregnancies. Dichorionic twins demonstrated higher VD levels than monochorionic twins, which can likely be attributed to greater functional placental mass, a reduced burden of placental complications, and relatively lower nutritional depletion resulting from more stable maternal hemodynamics. This study enhances the understanding of VD levels in women with twin pregnancies during mid-pregnancy under different clinical conditions and lays a foundation for future clinical pregnancy management. VD correlates with sPTB but not neonatal weight (1), consistent with singleton findings (16–18). The observed lack of VD-HDP/GDM association in twins contrasts with some studies (11, 12), potentially due to ethnic or environmental factors.

Prior studies suggest that VD prevents sPTB via modulating oxidative stress, gene expression, and immune regulation (26–30). This study highlights mid-pregnancy as the critical window for VD monitoring in high-risk twin pregnancies, which have higher PTB rates than singletons. Obstetricians should commence monitoring maternal VD levels from early pregnancy to allow adequate time for preventive measures aimed at reducing sPTB risk. We propose that this identification of mid-pregnancy as a “critical window” is closely linked to key processes uniquely characteristic of this gestational period—including immune modulation, placental development, and vascular remodeling—all of which are further amplified in the context of twin pregnancies: From an immunological tolerance perspective, the mid-pregnancy maternal–fetal interface is characterized by the establishment of an immunosuppressive environment that maintains a balance between immune tolerance and controlled inflammation to support normal fetal development (31). VD acts as an immunomodulator; deficiency during this critical period may disrupt this equilibrium, leading to the excessive activation of inflammatory pathways and heightened inflammatory responses (32). This process promotes calcium release within uterine myometrial cells (33), thereby triggering uterine contractions. By contrast, the immunotolerant state in late pregnancy is already well-established, and inflammatory pathways may have been primed. Consequently, VD supplementation during this later stage is unlikely to reverse entrenched inflammatory processes or reduce the risk of sPTB. From the perspective of placental development and functional maturation, the placental vasculature undergoes gradual maturation during mid-pregnancy, with improved endothelial cell function and extensive remodeling of the uterine spiral arteries (34). VD influences trophoblast cell differentiation and modulates placental angiogenesis through vascular endothelial growth factor (29). VD deficiency during this critical period may lead to reduced placental vascular branching density, impaired vascular endothelial function, and uteroplacental insufficiency, thereby contributing to sPTB. By the third trimester, placental development is complete and vascular remodeling has concluded. Hence, VD supplementation at this late stage is unlikely to reverse established placental defects and pathological changes. From the perspective of the unique physiology of twin pregnancies, rapid fetal growth after mid-pregnancy, coupled with a considerable expansion in uterine volume, readily heightens the contractile sensitivity of uterine smooth muscle. Furthermore, the placenta must undergo accelerated growth to meet the demands of two fetuses. VD deficiency during this critical period promotes increased calcium release within myometrial cells (33), thereby elevating uterine contractility susceptibility. It also impairs normal trophoblast proliferation and differentiation, ultimately compromising placental compensatory capacity. Collectively, these factors contribute to the establishment of a chronic, subclinical state of inflammation and ischemia at the maternal–fetal interface by early late pregnancy. Once established, this condition initiates an irreversible pathophysiological trajectory that may ultimately lead to sPTB by activating inflammatory pathways within the lower uterine segment or fetal membranes.

In contrast to sPTB, which is predominantly driven by biological mechanisms, iPTB is an iatrogenic decision motivated by clinical complications and demonstrates no clear association with VD levels. In cases of severe gestational complications, medical intervention—that is, the termination of pregnancy—is often the only recourse to prevent more serious outcomes such as stillbirth or intrauterine fetal demise. The pathophysiological drivers underlying these conditions are exceedingly powerful, far beyond what the immunomodulatory or other protective effects of VD could counterbalance. This specificity of association further reinforces the conclusion that VD deficiency in mid-pregnancy represents a modifiable risk factor specifically for sPTB.

The decision to employ RCS for threshold analysis was based on three primary reasons: First, preliminary logistic regression analysis indicated that VD deficiency was associated with sPTB, whereas sufficient and insufficient levels showed no significant association, suggesting a potential non-linear, saturation-dependent protective effect of VD. Second, existing VD classification criteria are largely based on singleton pregnancies, whilst twin pregnancy represents a distinct metabolic state; using RCS, we were able to identify a specific inflection point tailored to twin pregnancies. Third, RCS provides an intuitive visualization of risk trends, thereby offering more clinically informative and persuasive evidence than mere *p*-*values* or odds ratios.

The RCS model proposes a VD threshold of 25.06 ng/mL as a potential risk factor for sPTB in this single-center study; however, this finding remains exploratory and requires validation in external cohorts prior to clinical application. Future multicenter, large-sample research should explore VD's ethnic heterogeneity, establish optimal twin pregnancy VD threshold via prospective studies, and assess long-term maternal/neonatal impacts through basic research. RCS modeling revealed a non-linear relationship between mid-pregnancy VD concentrations and sPTB incidence (*p* for non-linearity < 0.05). Specifically, the protective effect of VD plateaued above a critical threshold, with no further reduction in sPTB risk being observed at higher concentrations. The analysis revealed that elevated late pregnancy VD levels had a limited effect on reducing the risk of sPTB, further supporting the conclusion that mid-pregnancy is the critical window period for preventing sPTB. Nevertheless, routine testing and supplementation remain necessary for pregnant women with VD deficiency/insufficiency. Future large-scale prospective cohort studies with longitudinal monitoring of VD are needed to elucidate its temporal dynamics and dose–response relationship.

We found no significant association between maternal VD levels during mid-pregnancy and neonatal birth weight, a finding consistent with several existing studies (1, 35). We propose that, in addition to gestational age at delivery, maternal genetic factors, and gestational weight gain rate, the primary determinants of neonatal birth weight in twin pregnancies include chorionicity, maternal HDP, and fetal sex combinations. Notably, neonates from dichorionic pregnancies exhibited higher birth weights compared to those from monochorionic pregnancies, aligning with the results reported in multiple prior studies (36, 37). The study demonstrated that male–male twin pairs had significantly greater neonatal birth weights than female–female twin pairs (*p* < 0.05), whereas no significant difference was detected between male–female and female–female pairs. We hypothesize that this phenomenon may result from antagonistic interactions between male and female hormones, and further research is required to elucidate the specific molecular mechanisms.

This study possesses several innovative aspects. Rather than solely focusing on the association between VD levels at a single time point and outcomes, we compared changes between mid- and late pregnancy, identifying the core conclusion that the “mid-pregnancy period is the critical window.” Moving beyond the question of whether there is an association, we addressed the clinically more significant question of when it is most relevant. This advances research on VD in twin pregnancies into a more refined and time-sensitive phase. The findings suggest that supplementing VD to sufficient levels in late pregnancy offers limited benefit. This indicates that for twin pregnancies, VD intervention must consider both dosage and timing, with the timing of intervention potentially being more critical than the dosage itself. This provides crucial timing evidence for future clinical trials and guidelines, suggesting that prophylactic supplementation should be initiated earlier, in early pregnancy or even pre-pregnancy. Furthermore, the data from the RCS curves indicate that findings from singleton pregnancy studies cannot be directly extrapolated to twin pregnancies, which may require their own specific risk thresholds. This lays a methodological foundation for future larger-scale, twin-specific research.

This study also has several limitations. Firstly, we lack information on the use of VD supplements. The research team conducted follow-up surveys on a subset of the enrolled participants regarding the use of VD supplements during pregnancy. The results indicated that all respondents had taken oral VD supplements during gestation, with the dosage adjusted based on serum VD levels. However, most respondents exhibited non-standardized medication practices. Given the partial absence of supplement usage data and the irregular supplementation patterns observed, the study ultimately did not incorporate supplement use into the formal analysis. Although maternal serum VD levels provide a more objective measure, it is important to acknowledge that this omission introduces limitations: specifically, the inability to evaluate the effectiveness of oral VD supplementation and the difficulty in defining clear intervention strategies. Consequently, the study cannot provide robust clinical guidance on supplementation protocols—including mode and dosage—specifically tailored for twin pregnancies. Secondly, this was a retrospective study conducted at a single tertiary medical center, which may limit the generalizability of the findings. Our patient population may not be representative of pregnant individuals from broader geographical regions and diverse socioeconomic backgrounds. The single-center nature of this study could have introduced unavoidable selection bias. Therefore, the relevant conclusions of this study require further validation in multicentre, prospective investigations. Finally, an issue arises from the partial missing data in late pregnancy. We did not opt to directly delete cases with missing data, as this would have resulted in a loss of sample size and potentially introduced more severe bias. Although sensitivity analysis and multiple imputation were performed—and produced consistent results, thereby strengthening the robustness of the conclusions to some extent—we cannot entirely rule out potential bias introduced by missing data. Furthermore, due to missing data and sample size limitations, certain subgroups in the complete-case analysis may have had insufficient sample sizes, and some comparisons might not have reached statistical significance. We therefore wish to emphasize the paramount importance of maintaining data completeness in future studies.

## Data Availability

The raw data supporting the conclusions of this article will be made available by the corresponding author upon request.
