# Supplementary material for: Longitudinal changes in vitamin D during twin pregnancy: association with maternal–neonatal outcomes
Source: Front Nutr. 2025 Nov 17;12:1667723. doi: 10.3389/fnut.2025.1667723 (PMC12665537; doi:10.3389/fnut.2025.1667723)
Supplement: Supplementary file 1 [file Table_1.docx]

Supplementary Material

**Supplementary Table 1.** Full results of multivariable logistic regression analysis for associations between confounding factors, vitamin D levels and spontaneous preterm birth

| Variable | Adjusted OR^a^ | 95% CI | *p*-Value |
| --- | --- | --- | --- |
| Vitamin D Levels |  |  |  |
| Sufficient (≥30 ng/mL) | 1.000 |  |  |
| Insufficient(20–30ng/mL) | 0.708 | 0.374 – 1.338 | 0.287 |
| Deficient (<20 ng/mL) | 2.190 | 1.066 – 4.499 | **0.033** |
| Employment Status |  |  |  |
| Employed | 1.000 |  |  |
| Unemployed | 0.722 | 0.387 – 1.346 | 0.306 |
| Chorionicity |  |  |  |
| Dichorionic | 1.000 |  |  |
| Monochorionic | 0.971 | 0.493 – 1.911 | 0.932 |
| Conception Method |  |  |  |
| Assisted Reproduction | 1.000 |  |  |
| Natural Conception | 0.935 | 0.507 – 1.726 | 0.830 |
| Parity |  |  |  |
| Nulliparous (0) | 1.000 |  |  |
| Multiparous (≥1) | 0.497 | 0.198 – 1.243 | 0.135 |
| Residence |  |  |  |
| Urban | 1.000 |  |  |
| Rural | 1.835 | 0.764 – 4.406 | 0.174 |
| Pre-pregnancy BMI(kg/m^2^) | 1.012 | 0.941 – 1.088 | 0.748 |
| Age(years) | 0.980 | 0.910 – 1.055 | 0.587 |

^a^Analyses were adjusted for all other variables listed in the table.

**Supplementary Table 2.** Demographic and clinical characteristics, stratified by preterm birth subtype

| Variable | sPTB  (n = 113) | iPTB  (n = 61) | Term Birth  (n = 150) | *p-*Value |
| --- | --- | --- | --- | --- |
| Age, years | 32.06 ± 3.72 | 32.95 ± 4.35 | 32.50 ± 3.65 | 0.328 |
| Advanced maternal age (≥35 years), n (%) | 30 (26.55) | 20 (32.79) | 49 (32.67) | 0.519 |
| Employed, n (%) | 85 (75.22) | 47 (77.05) | 119 (79.33) | 0.729 |
| Dichorionic twins, n (%) | 83 (73.45) | 52 (85.25) | 117 (78.00) | 0.202 |
| Assisted reproduction, n (%) | 59 (52.21) | 43 (70.49) | 86 (58.00) | 0.065 |
| Urban residence, n (%) | 104 (92.04) | 55 (90.16) | 132 (88.00) | 0.561 |
| Primiparity, n (%) | 98 (86.73) | 53 (86.89) | 140 (93.33) | 0.151 |
| Pre-pregnancy BMI, kg/m^2^ | 23.09 ± 3.63 | 23.33 ± 3.86 | 22.93 ± 3.34 | 0.765 |
| Pre-pregnancy BMI < 18.5 kg/m^2^, n (%) | 8 (7.08) | 1 (1.64) | 12 (8.00) | 0.223 |
| Vitamin D, ng/mL | 23.46 ± 8.92 * | 26.59 ± 7.97 | 25.68 ± 7.81 | **0.028** |
| Vitamin D Status |  |  |  |  |
| Sufficient (≥30 ng/mL), n (%) | 25 (22.12) | 21 (34.43) | 37 (24.67) | **0.001** |
| Insufficient (20–30 ng/mL), n (%) | 42 (37.17) | 26 (42.62) | 84 (56.00) |  |
| Deficient (<20 ng/mL), n (%) | 46 (40.71) | 14 (22.95) | 29 (19.33) |  |
| Sampling Season |  |  |  | 0.724 |
| Summer/Autumn, n (%) | 54 (47.79) | 33 (54.10) | 74 (49.33) |  |
| Winter/Spring, n (%) | 59 (52.21) | 28 (45.90) | 76 (50.67) |  |

* *p* = 0.018 vs. term birth group (LSD post hoc test).

**Supplementary Table 3.** Analysis of the association between vitamin D levels and spontaneous preterm birth in pregnant women with VD < 25.06 ng/mL

| Variable | Adjusted OR^a^ | 95% CI | *p*-Value |
| --- | --- | --- | --- |
| Vitamin D Levels | 0.874 | 0.805 – 0.949 | **0.001** |
| Employment Status |  |  |  |
| Employed | 1.000 |  |  |
| Unemployed | 1.552 | 0.628 – 3.832 | 0.341 |
| Chorionicity |  |  |  |
| Dichorionic | 1.000 |  |  |
| Monochorionic | 1.684 | 0.692 – 4.097 | 0.251 |
| Conception Method |  |  |  |
| Assisted Reproduction | 1.000 |  |  |
| Natural Conception | 0.899 | 0.371 – 2.177 | 0.813 |
| Parity |  |  |  |
| Nulliparous (0) | 1.000 |  |  |
| Multiparous (≥1) | 0.418 | 0.123 – 1.418 | 0.162 |
| Residence |  |  |  |
| Urban | 1.000 |  |  |
| Rural | 0.379 | 0.102 – 1.405 | 0.147 |
| Pre-pregnancy BMI(kg/m^2^) | 1.046 | 0.943 – 1.160 | 0.398 |
| Age(years) | 0.993 | 0.891 – 1.108 | 0.906 |

^a^Analyses were adjusted for all other variables listed in the table.

**Supplementary Table 4.** A comparison of spontaneous preterm birth rates between Groups 1 and 2 under varying conditions, alongside an analysis of the correlation between changes in vitamin D levels and spontaneous preterm birth.

| Condition | Group | Total  n, (%) | sPTB Rate (%) | *p*-Value | Crude OR  (95% CI) | *p*-Value | Adjusted OR^a^ (95% CI) | *p*-Value |
| --- | --- | --- | --- | --- | --- | --- | --- | --- |
| Conservative Assumption | Group 1 | 89  (67.94) | 49.44 | 0.337 | 1.000 |  | 1.000 |  |
|  | Group 2 | 42  (32.06) | 40.48 |  | 0.695  (0.331 – 0.142) | 0.338 | 1.134  (0.485 – 2.649) | 0.772 |
| Optimistic Assumption | Group 1 | 61  (46.56) | 44.26 | 0.622 | 1.000 |  | 1.000 |  |
|  | Group 2 | 70  (53.44) | 48.57 |  | 1.189  (0.597 – 2.369) | 0.622 | 1.472  (0.671 – 3.228) | 0.335 |
| Multiple Imputation | Group 1 | 79  (60.31) | 48.61 | 0.430 | 1.000 |  | 1.000 |  |
|  | Group 2 | 52  (39.69) | 43.46 |  | 0.810  (0.354 – 1.856) | 0.616 | 1.281  (0.497 – 3.304) | 0.606 |

^a^Analyses were adjusted for maternal age, chorionicity, conception method, employment status, parity, pre-pregnancy BMI and residence.

**Supplementary Table 5.** The effect of maternal vitamin D levels on neonatal anthropometric outcomes

| Variable | B | Beta | 95% CI | *p*-Value |
| --- | --- | --- | --- | --- |
| Head circumference | 0.007 | 0.031 | -0.009 – 0.023 | 0.406 |
| Chest circumference | -0.009 | -0.033 | -0.030 – 0.012 | 0.416 |
| Body length | 0.030 | 0.020 | -0.136 – 0.197 | 0.722 |

Analyses were adjusted for maternal age, maternal height, maternal pre-pregnancy BMI, maternal gestational weight gain rate, gestational age at delivery, chorionicity, maternal mid-pregnancy VD, GDM, HDP, and newborn sex.
